# Zhi-Kang-Yin formula attenuates high-fat diet-induced metabolic disorders through modulating gut microbiota-bile acids axis in mice

**DOI:** 10.1186/s13020-024-01021-w

**Published:** 2024-10-18

**Authors:** Yifan Li, Hao Wang, Xiaofang He, Weize Zhu, Yiyang Bao, Xinxin Gao, Wenjin Huang, Xinyu Ge, Wenjing Wei, Huan Zhang, Lili Sheng, Tao Zhang, Houkai Li

**Affiliations:** 1 School of Pharmacy, Shanghai University of Traditional Chinese Medicine, Shanghai, 201203 China; 2 Department of Liver Disease, The First Hospital of Hunan University of Chinese Medicine, Hunan, 410007 China

**Keywords:** Zhi-Kang-Yin, Metabolic disorder, Bile acids, Gut microbiota, Bile salt hydrolase

## Abstract

**Background:**

Metabolic disorders have become one of the global medical problems. Due to the complexity of its pathogenesis, there is still no effective treatment. Bile acids (BAs) and gut microbiota (GM) have been proved to be closely related to host metabolism, which could be important targets for metabolic disorders. Zhi-Kang-Yin (ZKY) is a traditional Chinese medicine (TCM) formula developed by the research team according to theory of TCM and has been shown to improve metabolism in clinic. However, the underlying mechanisms are unclear.

**Aim of the study:**

This study aimed to investigate the potential mechanisms of the beneficial effect of ZKY on metabolism.

**Methods:**

High-fat diet (HFD)-fed mice were treated with and without ZKY. The glucose and lipid metabolism-related indexes were measured. BA profile, GM composition and hepatic transcriptome were then investigated to analyze the changes of BAs, GM, and hepatic gene expression. Moreover, the relationship between GM and BAs was identified with functional gene quantification and ex vivo fermentation experiment.

**Results:**

ZKY reduced weight gain and lipid levels in both liver and serum, attenuated hepatic steatosis and improved glucose tolerance in HFD-fed mice. BA profile detection showed that ZKY changed the composition of BAs and increased the proportion of unconjugated BAs and non-12-OH BAs. Hepatic transcriptomic analysis revealed fatty acid metabolism and BA biosynthesis related pathways were regulated. In addition, ZKY significantly changed the structure of GM and upregulated the gene copy number of bacterial bile salt hydrolase. Meanwhile, ZKY directly promoted the growth of *Bifidobacterium*, which is a well-known bile salt hydrolase**-**producing genus. The ex vivo co-culture experiment with gut microbiota and BAs demonstrated that the changes of BAs profile in ZKY group were mediated by ZKY-shifted GM, which led to increased expression of genes associated with fatty acid degradation in the liver.

**Conclusion:**

Our study indicated that the effect of ZKY on improving metabolism is associated with the modulation of GM-BAs axis, especially, by upregulating the abundance of bile salt hydrolase-expression bacteria and increasing the levels of unconjugated BAs. This study indicates that GM-BAs axis might be an important pathway for improving metabolic disorders by ZKY.

**Supplementary Information:**

The online version contains supplementary material available at 10.1186/s13020-024-01021-w.

## Introduction

The increasing incidence of metabolic disorders, and related diseases including obesity, type 2 diabetes mellitus (T2DM), hyperlipidemia and metabolic dysfunction-associated fatty liver disease (MAFLD), poses a significant burden on global health. From 2000 to 2019, the prevalence of metabolic diseases has generally increased globally. But despite improvements in health care, mortality rates for T2DM and obesity have not declined [1]. Therefore, it is important to identify the pathogenesis of metabolic diseases and explore potential medical interventions. In addition to genetic factors, the main risk for metabolic syndrome is considered to be the disorder of glucose and lipid metabolism caused by unhealthy diet and lifestyle [2–4]. However, due to the complex pathogenesis of metabolic disorders, there is still a lack of effective treatment [3]. Therefore, it’s important to explore the specific factors affecting metabolic disorders and develop new therapeutic methods.

Bile acids (BAs) are among the main constituents of bile, which contribute to the dissolution and digestion of the dietary lipids [5]. In addition, BAs have also been reported to regulate energy, lipid and cholesterol metabolism through receptors like FXR (farnesoid X receptor) [6, 7]. BAs are synthesized by cholesterol in hepatocytes under the catalysis of a series of enzymes and most of them will be conjugated to glycine (G) or taurine (T) to form conjugated BAs. In intestinal, bacterial enzymes like bile salt hydrolase (BSH) and 7α-dehydroxylase can convert primary bile acids synthesized in liver into secondary bile acids by deconjugation or 7α-dehydroxylation, affecting the composition and balance of BAs [8]. There have been several studies suggest that some secondary BAs such as hyodeoxycholic acid (HDCA) and ursodeoxycholic acid (UDCA) can improve host lipid metabolism through multiple pathways. For example, HDCA has been reported to improve lipid metabolism and alleviate MAFLD by inhibiting intestinal FXR and upregulating hepatic CYP7B1, or activating PPARα signaling pathways [9, 10]. These studies suggests that BAs are important targets for the treatment of metabolic disorders.

Previous studies have shown that gut microbiota (GM) composition is also closely related to host metabolism [11]. For example, compared with healthy people, MAFLD patients had more gram-negative bacteria and less gram-positive bacteria in their feces [12]. More and more evidence also shows that the imbalance of GM is an important factor in inducing obesity and related metabolic diseases [13]. GM imbalance can affect the production of BAs and short-chain fatty acids and the permeability of intestine [14]. In addition, GM could directly regulate AMPK expression and other ways to regulate the host fatty acid synthesis, oxidative metabolism, insulin sensitivity, carbohydrate metabolism and other processes, which ultimately affect the host metabolism homeostasis [13].

Traditional Chinese Medicine (TCM) is widely considered to have the advantage of regulating multiple targets and pathways simultaneously, and many studies have confirmed the efficacy of TCM in treating metabolic diseases [15–19]. Zhi-Kang-Yin (ZKY) is a traditional Chinese medicine herbal formula developed by the research team according to the theory of TCM in “*Dan Xi Zhi Fa Xin Yao*” written by Zhu Danxi in the Yuan Dynasty, which mentioned “obese people often have phlegm-dampness syndrome”. ZKY is composed of six herbs: *Radix Bupleuri*, *Curcumae Radix, Crataegi Fructus, Salviae Miltiorrhizae Radix Et Rhizoma, Aurantii Fructus*, and *Alismatis Rhizoma*, which has the effect of dispelling dampness and resolving phlegm*.* In previous studies, ZKY has been shown to improve liver function and lipid levels in clinical patients with MAFLD [20] and ameliorate diet-induced obesity in rats [21]. However, the specific mechanism of ZKY improving metabolic disorders is still unclear and remains to be explored.

In this study, we observed the therapeutic effect of ZKY on ameliorating high-fat diet (HFD)-induced metabolic disorders. Subsequently, we investigated the changes of GM and BA profiles in serum and liver tissue of mice as well as the changes of hepatic gene expression. Finally, we validated the relationship between GM and BAs after ZKY intervention through functional gene quantification and ex vivo co-culture. Our study shows that ZKY can alleviate metabolic disorder phenotypes including body weight gain, hepatic steatosis, and impaired glucose homeostasis. These effects could be attributed to the regulation of the GM-BA axis, which leads to the increased abundance of BSH-producing bacteria and increased proportion of unconjugated BAs.

## Materials and methods

### Chemical materials and reagents

The following chemical materials were used in the study: Methanol (purity ≥ 99.9%, M116118), formic acid (purity ≥ 99%, F301957) and obeticholic acid (OCA, purity ≥ 98%, I193491) were purchased from Aladdin (Shanghai, China); paraformaldehyde (G1101, Servicebio, Wuhan, China); hematoxylin and eosin staining kit (C0105S, Beyotime, China); total cholesterol (TC), triglyceride (TG), alanine aminotransferase (ALT), aspartate aminotransferase (AST), low density lipoprotein-cholesterol (LDL-c) and high density lipoprotein-cholesterol (HDL-c) assay kit (A111-1-1, A110-1-1, C009-2-1, C010-2-1, A113-1-1, A112-1-1, Nanjing Jiancheng Bioengineering institute, China) and all RT-qPCR primers (Biosune, Shanghai, China).

### Preparation of ZKY decoction and chemical components identification

The details of preparation of ZKY decoction and the component identification was shown in supplemental material and Fig. S1 and Table S1.

### Animals

Four-week-old male C57BL/6 mice were randomly divided into 5 groups after adaptive feeding with chow diet for a week. Mice in control group (Con, *n* = 6) were fed with chow diet and mice in all other 4 groups were fed with HFD (60% fat, #D12492, Research Diets) for 15 weeks. The formulation of HFD is provided in Table S2. In addition to 15-week HFD feeding, mice in the ZKY-L group and ZKY-H group were given low-dose (11.25 g/kg) and high-dose (22.5 g/kg) ZKY respectively by intragastric administration for 11 weeks starting from the fourth week. What’s more, mice in OCA group (*n* = 6) were given 0.04% OCA mixed in HFD as positive control group. The dose of OCA was selected based on previous publications [22–25]. For the HFD, ZKY-L and ZKY-H group, 8 mice were distributed to these groups respectively. All groups were provided with normal drinking water and all mice were weighed once a week. This animal experiment was approved by the Animal Ethical Committee of Shanghai University of Traditional Chinese Medicine (Approval No. PZSHUTCM2311210007, Date: 14th Nov. 2023).

### Glucose tolerance test

Intraperitoneal glucose tolerance test (IPGTT) was performed in mice after fasting for 12 h. After an intraperitoneal injection of glucose solution at a dosage of 1 g/kg body weight, the blood glucose level of tail vein was measured with glucose analyzer at 0, 15, 30, 60, 90 and 120 min. Six mice in each group were randomly selected for IPGTT and the area under glycemic curve (AUC) was calculated after the test.

### Histological examination

Liver tissue and epididymal white adipose tissue were fixed with 4% paraformaldehyde, embedded in paraffin and sectioned to make microscope slides. The tissue slides were stained with hematoxylin and eosin. The degree of hepatic steatosis was then evaluated according to the previous publication [26].

### Detection of liver and serum biochemical indexes

The hepatic lipids were extracted and purified by Folch method [26]. The hepatic TC and TG levels and serum TC, TG, ALT, AST, LDL-c and HDL-c concentrations were all measured according to the instructions of corresponding assay kits.

### 16S rRNA sequencing

The sequencing was performed according to our published study [27]. The bacterial genomic DNA samples was extracted from cecal content by TIANamp Stool DNA Kit (TIANGEN). The V3-V4 variable region of 16S rRNA gene was amplified using the primers 338F (5′-ACTCCTACGGGAGGCAGCAG-3′) and 806R (5′-GGACTACHVGGGTWTCTAAT-3′) in PCR. The amplified gene fragment was sequenced by the Illumina Miseq PE300 on the Majorbio Cloud Platform and clustered to similar OTUs (≥ 97% similarity) by UPARSE and the chimeric sequences were eliminated, based on the Silva 16S rRNA database (SSU138).

### Targeted metabolome profiling of BAs

BAs quantification was performed by Metabo-Profile Biotechnology (Shanghai) Co., LTD. Different bile acids and isotope internal standards were dissolved in methanol to obtain a stock solution with a concentration of 5 mM. The stock solution was mixed during use, and then diluted stepwise to different concentration points in a bile acid-free matrix. In addition, bile acid standard solutions with concentrations of 1500, 150 and 5 nM were prepared using bile acid-free matrix as quality control samples. All standard solutions and quality control samples were prepared to have the same concentration Internal standard within (with GCA-d4, TCA-d4, TCDCA-d9, UDCA-d4, CA-d4, GCDCA-d4, GDCA-d4, DCA-d4, LCA-d4 and β-CA-d5 all at the concentration of 150 nM).

For serum samples, the serum and standard solution were mixed with acetonitrile: methanol (v/v = 80:20), which contained internal standard, followed by extraction of bile acids using a centrifuge at 10 ℃ and 1500 RPM for 15 min. The resulting supernatant is then transferred to a 96-well plate and freeze-dried in a freeze-dryer. The dried samples, standard and quality control samples were then redissolved with a mixture of 1:1 acetonitrile/methanol (80/20) and ultrapure water. For liver tissue samples, about 10mg of tissue was added to the centrifugal tube, and the grinding bead and ultra-pure water were added to homogenize. After homogenization, it was then centrifuged at 4 ℃ at 13,500 RPM for 20 min. The following procedure were as same as the serum samples. Finally, the supernatant was transferred to a 96-well plate for subsequent UPLC-MS/MS analysis with a volume of 5 μl.

### Hepatic transcriptomic analysis

Total RNA was extracted from liver tissue using TRIzol Reagent according to the manufacturer’s instructions. Then RNA quality was determined by 5300 Bioanalyzer (Agilent) and quantified using the ND-2000 (NanoDrop Technologies). The RNA-seq library was prepared following Illumina Stranded mRNA Prep, Ligation (San diego, CA) using 1 ug of total RNA and it was performed on NovaSeq X Plus platform (PE150). The raw paired end reads were trimmed, and quality controlled by FASTP. Then clean reads were aligned to reference genome with orientation mode using HISAT2 software. The expression level of each transcript was calculated according to the transcripts per million reads (TPM) method. RSEM was used to quantify gene abundances and differential expression analysis was performed using the DESeq2.

### Quantification of BSH

The quantification of BSH was based on the published study [28]. *Escherichia coli* TOP10 containing pUC57-Bsh plasmid was cultured overnight and then the plasmid was extracted by employing EndoFree Mini Plasmid Kit II (TIANGEN). The concentration of plasmids was determined by ultraviolet-spectrophotometer and converted to copy number. The plasmids were diluted to 1 × 10^10^ copies/μL and then further diluted in a tenfold gradient to make standard samples. Standard curve of the above samples was drawn by performing qPCR with specific primers (Forward: 5′-ATGGGCGGACTAGGATTACC-3′ and Reverse: 5′- TGCCACTCTCTGTCTGCATC-3′). The concentration of bacterial genomic DNA sample was then measured and amplified by qPCR. The Ct values of each sample were substituted into the standard curve and the copy number of BSH was then obtained.

### Bacteria growth experiment

*Bifidobacterium animalis, Bifidobacterium longum, Lactobacillus acidophilus*, and *Lactobacillus casei* were all obtained from ATCC. All strains were cultured in corresponding medium containing 0, 0.1, 1 and 10 mg/ml ZKY respectively (*n* = 3) at 37℃ under anaerobic conditions. The optical density (OD) at 600 nm of each sample was measured at 0, 3, 6, 12, 24, 36, 48 h through microplate reader to evaluate the growth situation of bacteria and the growth curve was then obtained.

### Ex vivo co-culture of bile acids with cecal bacteria

In co-culture experiment 1, the cecal contents of mice in Con, HFD and ZKY group were collected respectively, and cecal bacteria were then obtained by centrifugation. On the other hand, bile juice was collected from 4-week-old C57BL/6J wild type mice. Then, the cecal bacteria were cultured at 37℃ under anaerobic conditions in a sterilized mixture of bile juice and GB medium (BB: mGAM = 3.5:6.5), which is a medium optimized by our team [29]. The samples were collected at 0 and 48 h, and the supernatant was then collected for targeted metabolome profiling of bile acids after centrifugation. The pretreatment of the supernatant was similar to that of serum samples and the mixture of acetonitrile: methanol (v/v = 80:20) and the supernatant was centrifuged at 10℃ and 650 RPM for 20 min.

In co-culture experiment 2, the fresh stool of Con mice was obtained, and bacteria were collected. Half bacteria were co-cultured with bile juice as in the above experiment for 48 h and the supernatant of this group (ConB_BA) was collected. The other bacteria were cultured in GB medium containing 10 mg/ml ZKY for 48 h. Then, ZKY-altered GM were inoculated in GB medium (1:100 dilution), and co-cultured with bile juice for another 48 h. The supernatant of second group (ZKYB_BA) was also collected. After that, the supernatant of two groups was freeze-dried and redissolved in AML-12 medium.

### AML-12 cell line study

The mice AML-12 cells were seeded in 12-well plate. When the cells have grown to about 50%, they were divided into 4 groups, which were Con, Model, ConB_BA and ZKYB_BA. Cells in the latter three groups were given 250 uM palmitic acid and 500 uM oleic acid (PA/OA) to induce lipid accumulation. Cells in ConB_BA and ZKYB_BA group were given corresponding redissolved BA-containing supernatant, which was diluted in AML-12 medium with a ratio of 1:8 and has no effect on cell growth. The PA/OA and the supernatant were simultaneously used to treat cells at the beginning of experiment. After 48 h of culture, the cells were collected for further experiments.

### RNA isolation and real-time quantitative PCR (RT-qPCR)

The EZ-press RNA Purification Kit (EZBioscience, USA) was employed to extract total RNA from AML-12 cells. The cDNA was then obtained using 4 × Reverse Transcription Master Mix (EZBioscience). RT-qPCR was then performed using ChamQ SYBR qPCR Master Mix (Vazyme, China), and relative gene expression was analyzed by 2^−∆∆CT^ method. The genes and primer sequences used were provided in Table S3.

### Statistical analysis

GraphPad Prism 8 was employed for statistical analyses and all data were shown as mean ± standard error of mean (SEM) unless otherwise noted. Significant differences were assessed through Kruskal–Wallis H test or one-way ANOVA with Tukey’s multiple comparison. A *p value* < 0.05 was considered as significant.

## Results

### ZKY alleviated HFD-induced metabolic disorder in mice

**Fig. 1:**
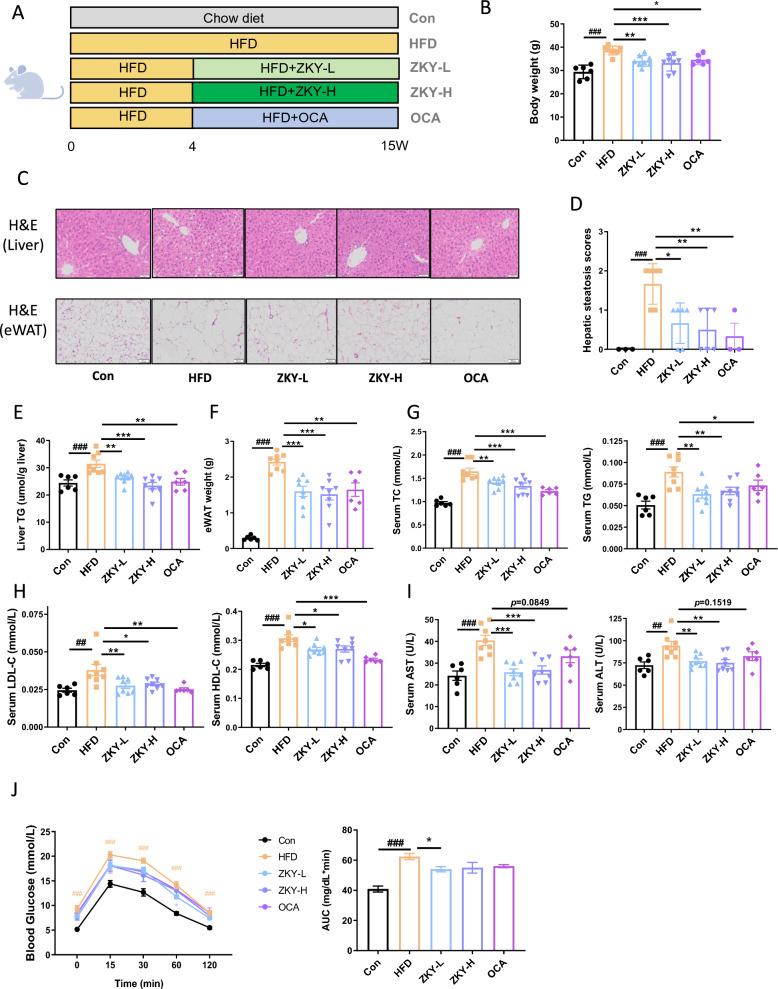
ZKY alleviated HFD-induced metabolic disorder in mice. **A** Diagram shows the experimental design. **B** The final body weight at the end of experiment. **C** Representative images of liver tissues and eWAT (epididymal WAT). **D** Hepatic steatosis scores (*n* = 3 in Con and OCA and *n* = 6 in other groups). **E** Liver TG level. **F** eWAT weight. **G** Serum TC and TG level. **H** Serum LDL-C and HDL-C level. **I** Serum AST and ALT level. **J** Glucose Tolerance Test (GTT) and Area Under Curve (AUC) (*n* = 6). All data are shown as the mean ± SEM (*n* = 6–8); difference between groups are measured by one-way ANOVA followed by Tukey’s multiple comparison. ^##^*p* < 0.01, ^###^*p* < 0.001 (HFD *vs.* Con); *p < 0.05, **p < 0.01, ***p < 0.001 (ZKY *vs.* HFD)

To confirm the impact of ZKY on HFD-induced metabolic disorder, 4-week-old mice were fed with HFD for 15 weeks. Among them, the ZKY-L group, ZKY-H group and OCA group were treated with different doses of ZKY and OCA respectively (Fig. 1A). The weight gain due to HFD was significantly reduced by both concentrations of ZKY, which showed a better effect than OCA (Fig. 1B). At the same time, there was no significant difference in energy intake between the HFD group and the ZKY intervention group (Fig. S2), which indicated that the weight loss was not the result of reduced appetite of the mice in ZKY group.

H&E staining of liver tissue and the liver TG level showed that HFD feeding caused severe hepatic steatosis, which was alleviated by ZKY intervention (Fig. 1C–E). H&E staining and weight of eWAT also showed the role of ZKY in reducing lipid accumulation (Fig. 1C and F). Additionally, ZKY also significantly reduced several serum biochemical indexes compared with HFD group, which included serum TC, TG, LDL-c, HDL-c, ALT and AST levels, especially for serum ALT and AST which did not significantly decline in OCA group (Fig. 1G–I). All these results showed an improvement of metabolism. What’s more, glucose tolerance test (GTT) showed that only ZKY at low doses demonstrated a certain effect in improving glucose homeostasis (Fig. 1J).

### ZKY regulated BA profile in serum and liver

**Fig. 2:**
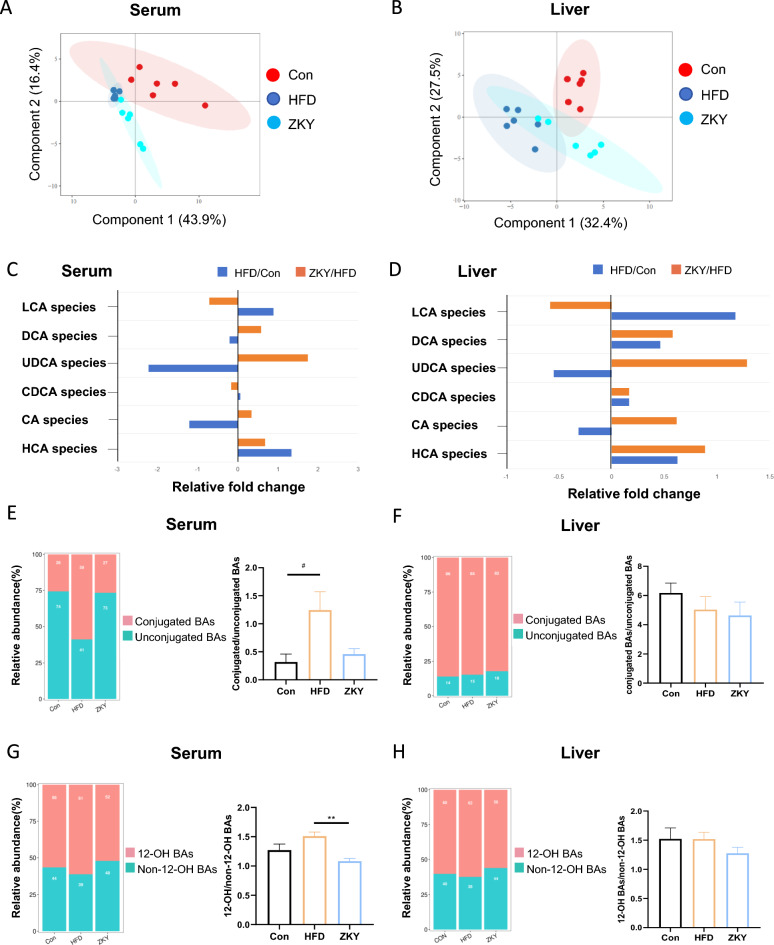
ZKY changed the composition of bile acid profile both in serum and liver. **A**, **B** Partial least squares Discriminant Analysis (PLS-DA) plot of BA profile in serum and liver. **C**, **D** Fold changes of 6 BA species in serum and liver in HFD than Con and ZKY than HFD. **E**, **F** The relative abundance of conjugated BAs and unconjugated BAs and the ratio between these two in serum and liver. **G**, **H** The relative abundance of 12-OH BAs and non-12-OH BAs and the ratio between these two in serum and liver. All data are shown as the mean ± SEM (*n* = 5–6); difference between groups are measured by Kruskal–Wallis test followed by Tukey’s multiple comparison. ^#^*p* < 0.05 (HFD *vs.* Con); *p < 0.05, **p < 0.01 (ZKY *vs*. HFD)

To identify the effect of ZKY on bile acid metabolism, the BA composition in serum and liver was analyzed after targeted metabolome profiling. First of all, the PLS-DA plot indicated that both serum and liver BA profiles showed a certain degree of separation among the three groups (Fig. 2A, B). In both BA profiles, UDCA species and CA species showed a decreasing trend in Con group compared to HFD group but increased in ZKY group compared to HFD group, while the LCA species demonstrated an opposite trend. In addition, HCA species increased in HFD compared with Con and further increased in ZKY group. The other BA species all showed different trends in two BA profiles (Fig. 2C, D). In serum BA profile, the proportion of unconjugated BAs significantly decreased in HFD group while increased in ZKY group (Fig. 2E). However, compared with Con group, the proportion mentioned above only showed a slight increasing trend in HFD and ZKY group in liver BA profile (Fig. 2F). Similarly, the ratio of 12-OH to non-12-OH BAs strikingly decreased in ZKY group compared to HFD group in serum BA profile while showed no significant difference among three groups in liver BA profile (Fig. 2G, H).

**Fig. 3:**
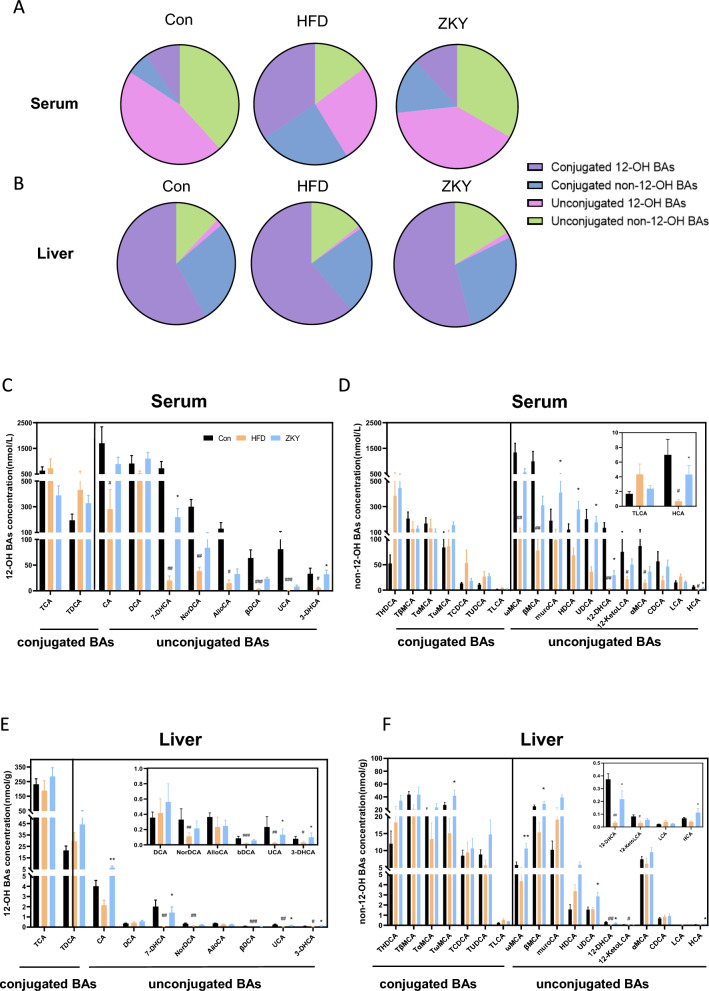
The effect of ZKY on specific BA concentration in serum and liver. **A**, **B** The percentage of the four types of BAs (conjugated 12-OH BAs, conjugated non-12-OH BAs, unconjugated 12-OH BAs and unconjugated non-12-OH BAs) in serum and liver. **C**, **D** The changes of individual BA concentration (nmol/L) in serum. **E**, **F** The changes of individual BA concentration (nmol/g) in liver. All data are shown as the mean ± SEM (*n* = 5–6); difference between groups are measured by Kruskal–Wallis test followed by Tukey’s multiple comparison. ^#^*p* < 0.05, ^##^*p* < 0.01, ^###^*p* < 0.001 (HFD *vs.* Con); *p < 0.05, **p < 0.01 (ZKY *vs.* HFD)

Next, we further divided bile acids into four different types based on whether they have a 12-OH and whether they are conjugated with taurine or glycine and calculated their ratios. In agreement with the previous conclusion, the unconjugated non-12-OH BAs in serum showed remarkable decreasing and increasing trends in the HFD group and ZKY group respectively, but similar trends could not be found in the liver BA profile (Fig. 3A, B). To investigate the changes of individual BAs, we selected bile acids with relatively high concentration in both serum and liver, divided them into four types mentioned above and analyzed their changes among three groups (Fig. 3C–F). In serum, compared with the HFD group, the 12-OH BAs that changed significantly in ZKY group included 7-DHCA and 3-DHCA, and there were more non-12-OH BAs significantly changed, including muroCA, HDCA, UDCA and 12-DHCA. In liver, among the 12-OH BAs, in addition to the bile acids mentioned above, CA and UCA were also significantly increased in the ZKY group. Besides, non-12-OH BAs significantly elevated included ωMCA, βMCA, UDCA and DHCA. Interestingly, whether in serum or liver, the bile acids that significantly increased in the ZKY group were basically unconjugated BAs, which means the increase in the levels of unconjugated BAs may be related to the efficacy of ZKY.

### ZKY significantly affected hepatic gene expression

**Fig. 4:**
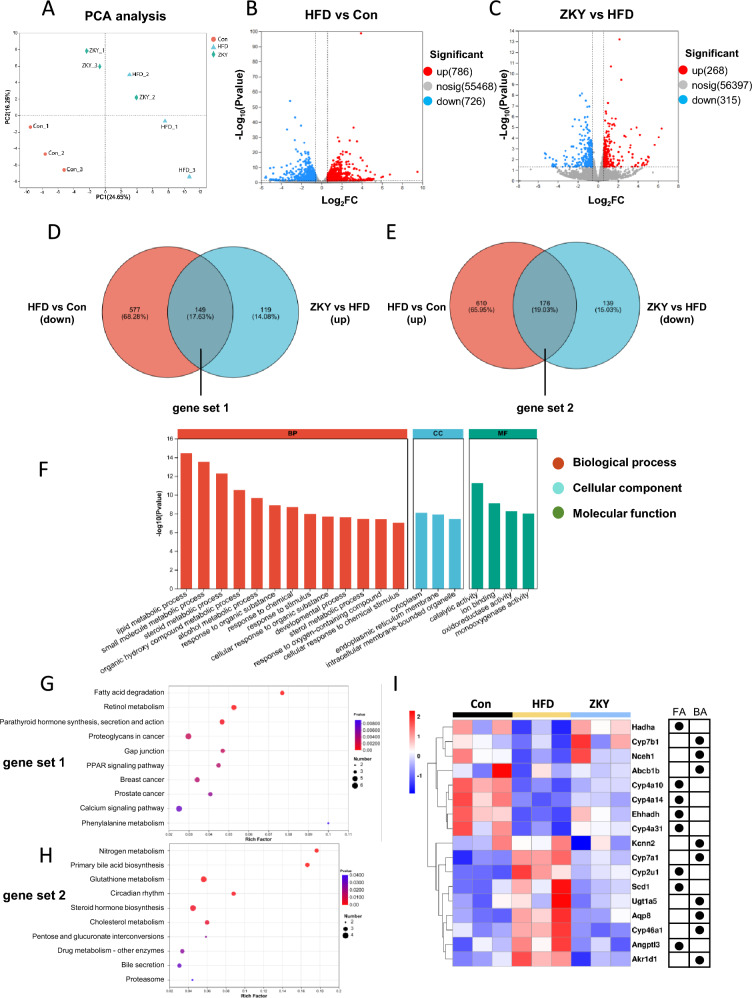
ZKY intervention altered gene expression profile of liver. **A** Principle component analysis of hepatic gene expression profile of three groups. **B** Volcano plot of DEGs between HFD and Con group. The screening criteria was FC > 1.5 and *p* value < 0.05. **C** Volcano plot of DEGs between ZKY and HFD group. The screening criteria was FC > 1.5 and *p* value < 0.05. **D** Venn diagram of genes significantly downregulated in HFD group compared to Con group and significantly upregulated in ZKY group compared to HFD group (gene set 1). **E** Venn diagram of genes significantly upregulated in HFD group compared to Con group and significantly downregulated in ZKY group compared to HFD group (gene set 2). **F** GO enrichment analysis of genes significantly reversed by ZKY (set1 + set2). **G** KEGG enrichment analysis of genes in set 1. The top 10 pathways with lowest *p* value was demonstrated. **H** KEGG enrichment analysis of genes in set 2. The top 10 pathways with lowest *p value* was demonstrated. **I** Heatmap of the genes, which related to fatty acid (FA) and bile acid (BA) metabolism pathway in **G**, **H** and significantly altered in ZKY group compared to HFD group, were shown

To further explore the mechanism by which ZKY improves metabolic disorders, RNAseq was conducted to search for potential target genes and signaling pathways. According to PCA analysis, both HFD and ZKY intervention caused significant changes in the hepatic gene expression profile, and at the PC1 level, the gene expression of ZKY group was more similar to that of Con group (Fig. 4A). The differentially expressed genes (DEGs) among the three groups were screened under the standard of *p value* < 0.05 and fold change (FC) > 1.5. Compared with Con group, 786 genes were upregulated in HFD group, and 726 genes were downregulated (Fig. 4B). Meanwhile, compared with HFD group, the number of significantly upregulated and downregulated genes in ZKY group was 268 and 315 respectively (Fig. 4C). Among these DEGs, 149 genes (gene set 1) were downregulated in HFD group while upregulated in ZKY group and 176 genes (gene set 2) demonstrated an opposite trend (Fig. 4D, E).

In addition, the gene ontology (GO) enrichment analysis was performed based on the 325 genes reversed by ZKY (Fig. 4F). Most notably, these genes were mostly enriched in lipid metabolism process in biological process, which was consistent with the effect of ZKY on improving lipid metabolism. The reversed genes were also enriched in endoplasmic reticulum membrane in cellular component, which is related to lipid synthesis, and catalytic activity and ion binding in molecular function. What’s more, Kyoto Encyclopedia of Genes and Genomes (KEGG) enrichment analysis demonstrated that genes in set 1 were closely related to fatty acid degradation and PPAR signaling pathway, which suggested that fatty acid oxidation was an important way for ZKY to improve lipid metabolism (Fig. 4G). On the other hand, genes in set 2 were connected to primary bile acid biosynthesis and bile secretion, which demonstrated that bile acid-related pathway might be suppressed by ZKY (Fig. 4H). Among the genes associated with these pathways, the expression levels of genes that differed significantly between the HFD and ZKY groups were shown in Fig. 4I, indicating the potential role of ZKY on regulating the expression of these target genes.

### ZKY regulated gut microbiota composition

**Fig. 5:**
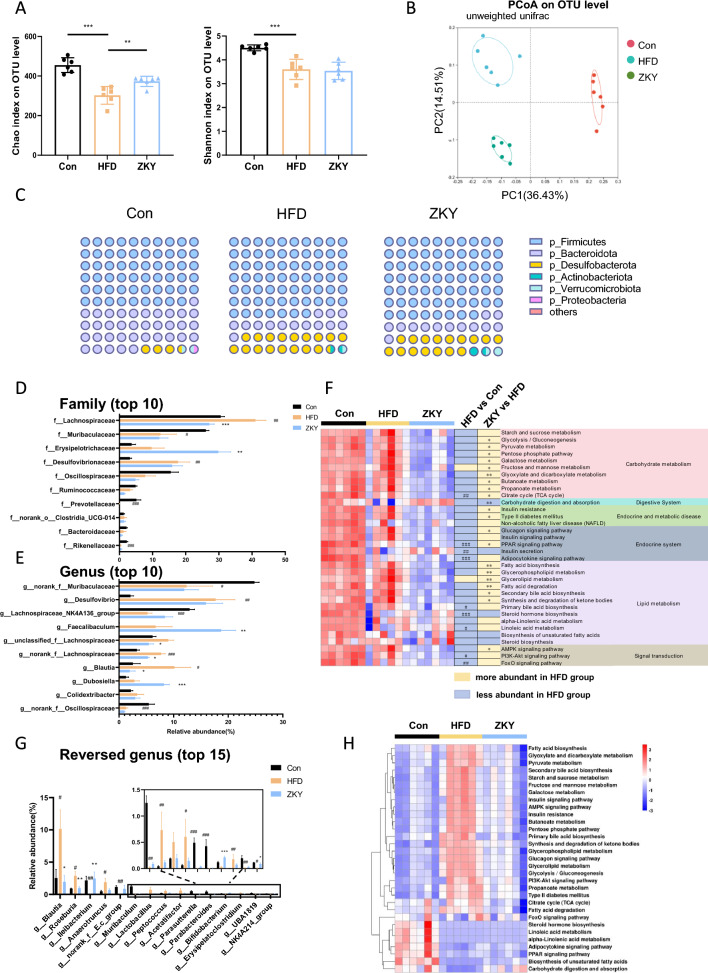
Mice treated with ZKY showed different GM composition from HFD-fed mice. **A** Chao index and Shannon index on OTU level. **B** Principal co-ordinates analysis (PCoA) plot based on unweighted unifrac on OTU level. **C** A dot plot shows the relative abundance of cecal bacteria at phylum level. Each dot represents an abundance of 1% of the total GM. **D** Relative abundance of top10 abundant family. **E** Relative abundance of top 10 abundant genus. **F** Heat map of changes of GM function associated with glucose and lipid metabolism KEGG pathways. **G** Relative abundance of top 15 co-altered genera in Con *vs.* HFD and HFD *vs.* ZKY (Fold change > 2 or < 0.5). **H** Heat map of functional changes of reversed genera with glucose and lipid metabolism related KEGG pathways. All data are shown as the mean ± SEM (*n* = 6); difference between groups are measured by Kruskal–Wallis test followed by Tukey’s multiple comparison. ^#^*p* < 0.05, ^##^*p* < 0.01, ^###^*p* < 0.001 (HFD *vs.* Con); *p < 0.05, **p < 0.01, ***p < 0.001 (ZKY *vs.* HFD)

To investigate the effect of ZKY on GM, the result of 16S rRNA gene sequencing was analyzed. Firstly, the feeding of HFD resulted in the significant decrease of Chao index and Shannon index, while the intervention of ZKY only had an increasing effect on Chao index, which means ZKY increased the richness but not the evenness of bacteria (Fig. 5A). Besides, Unweighted unifrac principal coordinate analysis (PCoA) on OTU level showed that both HFD feeding and ZKY intervention produced significant changes in the gut microbiota (Fig. 5B). Next, we further analyzed the composition of gut microbiota on different levels. On phylum level, the proportion of *p_Firmicutes* increased while which of *p_Bacteroidetes* decreased in HFD-feeding mice compared with Con group, and this trend did not change in ZKY group (Fig. 5C).

At family level, we analyzed the top 10 families in relative abundance (Fig. 5D). Compared with HFD group, the relative abundance of *f_Lachnospiraceae* in ZKY group markedly decreased, while that of *f_Erysipelotrichaceae* increased significantly. At genus level, the relative abundance of *g_Faecalibaculum* and *g_Dubosiella* from *f_Erysipelotrichaceae* in ZKY group showed a significant increasing trend, while *g_Blautia* from *f_Lachnospiraceae* decreased distinctly, which was consistent with the previous analysis results (Fig. 5E). In addition, we predicted the functional pathways of microbiota through PICRUSt2 analysis based on the 16S rRNA gene (Fig. 5F). We focused on the changes of KEGG level 3 pathways related to glucose and lipid metabolism. From the analysis result, it could be found that many pathways were significantly different in expression between the HFD and ZKY groups. However, only a small number of pathways such as Fructose and mannose metabolism, Carbohydrate digestion and absorption, Insulin secretion, Glycerolipid metabolism, Steroid hormone biosynthesis, Biosynthesis of unsaturated fatty acids, and Steroid biosynthesis showed reversed trends between HFD *vs.* Con and ZKY *vs.* HFD.

To further explore the co-altered bacterial genera in HFD *vs.* Con and ZKY *vs.* HFD, we screened the top 15 co-altered bacterial genera based on the changes of relative abundance (Fold change > 2 or < 0.5) (Fig. 5G) and the microbial functional predication showed that majority of pathways that related to glucose and lipid metabolism were upregulated in HFD group and reversed in ZKY group. Among these pathways, we found that the primary/secondary bile acid biosynthesis pathway were all significantly altered by ZKY, suggesting that these critical genera were closely related to bile acid metabolism (Fig. 5H).

### ZKY intervention upregulated BSH expression

**Fig. 6:**
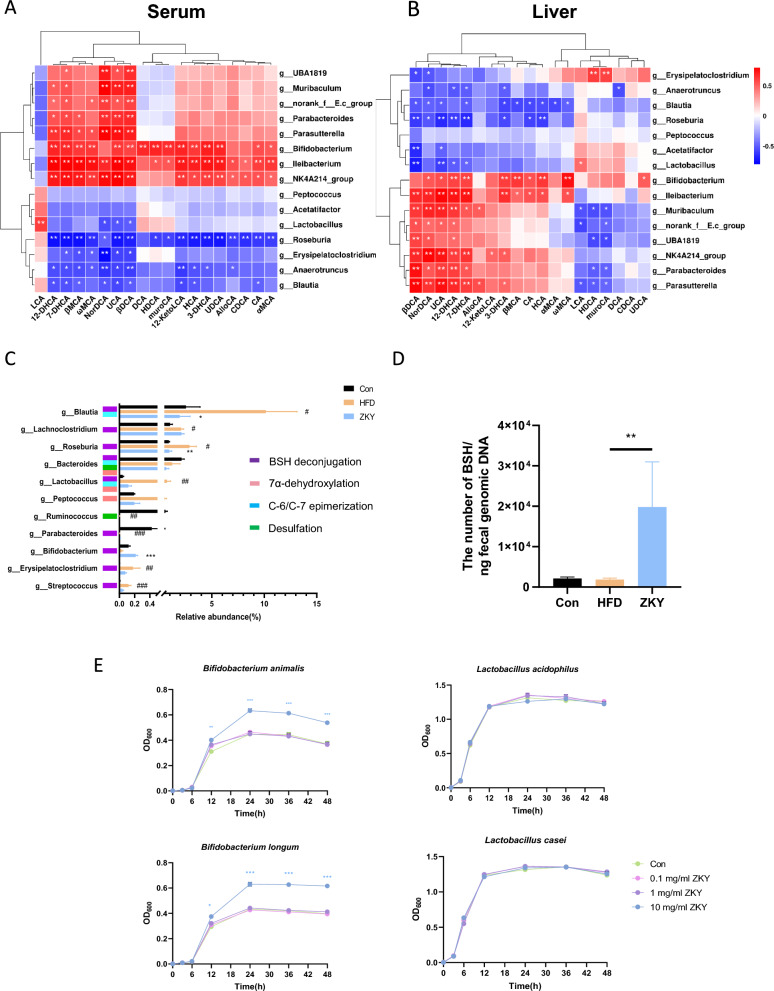
Regulation of ZKY on bile acid metabolism-related bacteria and BSH. **A**, **B** Spearman's correlation analysis between top 15 abundant genus reversed by ZKY and unconjugated non-12-OH BAs in serum and liver among three groups. **C** Relative abundance of BA metabolism-related genera (*n* = 6). **D** The copy number of BSH gene among three groups (*n* = 6). **E** Growth curves of four BSH-producing bacteria (*n* = 3). All data are shown as the mean ± SEM; difference between groups are measured by Kruskal–Wallis test or one-way ANOVA followed by Tukey’s multiple comparison. ^#^*p* < 0.05, ^##^*p* < 0.01, ^###^*p* < 0.001 (HFD *vs.* Con); *p < 0.05, **p < 0.01, ***p < 0.001 (ZKY *vs.* HFD or ZKY-containing group *vs.* Con group)

To explore the relationship between changes in gut microbiota and bile acids, the spearman correlation analysis of co-altered bacterial genera with unconjugated BAs in serum and liver was performed separately (Fig. 6A, B)*.* In serum, *g_Ilebacterium*, *g_Bifidobacterium* and *g_NK4A214 group* were significantly positively correlated with most unconjugated BAs, while *g_Roseburia, g_Blautia* and *g_Anaerotruncus* showed opposite trends. In the liver, similar significant trends could still be observed in *g_Bifidobacterium* and *g_Blautia.* Further, we analyzed the changes in main bacterial genera related to bile acid metabolism between the three groups (Fig. 6C). Compared with the HFD group, most bacteria related to bile acid metabolism showed a decreasing trend in ZKY group, only *g_Bifidobacterium* was significantly increased after ZKY intervention. Since the sequencing results showed only some of BA metabolism-related bacteria which has been reported, we speculated that other related bacteria, like *Clostridiuum* and *Enterococcus* might be classified as unknown or unclassified on genus level. In order to directly confirm the changing tendency of BSH between groups, we measured the expression of BSH (Fig. 6D). The quantification result showed that the copy number of BSH was significantly increased in the feces of ZKY group, which means that ZKY intervention can indeed promote the expression of BSH.

Meanwhile, in order to explore whether ZKY could directly promote the growth of BSH-producing bacteria, we performed ZKY and bacteria co-culture experiment. Genera from *Bifidobacterium* and *Lactobacillus* are well-known bacteria for containing BSH gene [30, 31]. In addition, 16S rRNA sequencing result showed that ZKY increased the relative abundance of *Bifidobacterium*. Thus, *Bifidobacterium animalis, Bifidobacterium longum, Lactobacillus acidophilus*, and *Lactobacillus casei* were used in this study to show whether ZKY could stimulate the growth of these bacteria (Fig. 6E). The result indicated that 10 mg/ml of ZKY could promote the growth of both *Bifidobacterium animalis* and *Bifidobacterium longum,* which was consistent with the result of 16S rRNA sequencing, while have no effect on the growth of *Lactobacillus acidophilus* or *Lactobacillus casei*. These findings suggest ZKY could increase the expression of BSH partially by promoting the growth of BSH-producing bacteria.

### ZKY-intervened GM increased the ratio of unconjugated bile acids and regulated fatty acid metabolism related genes

**Fig. 7:**
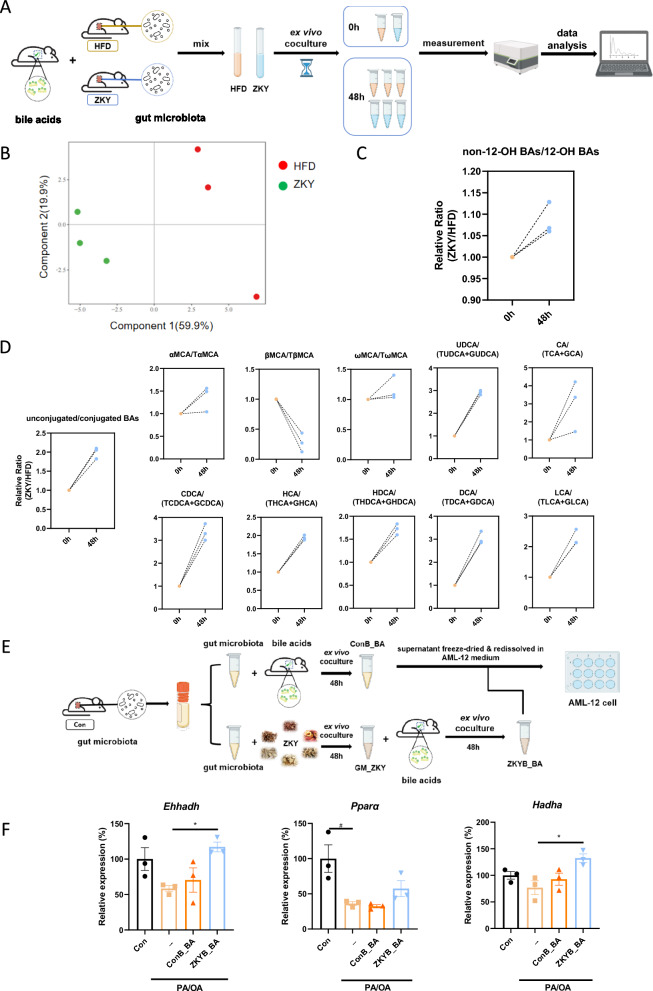
Effect of ZKY-altered GM on bile acid metabolism. **A** Design of ex vivo co-culture experiment 1. The cecal bacteria of HFD and ZKY mice was obtained and cultured in GB medium containing fresh bile juice collected from wild type mice. After mixing cecal bacteria with the medium, 0 h samples from both groups were collected immediately, and then each group was divided into three centrifuge tubes for 48 h culture (*n* = 3). **B** PLS-DA plot of BA profile at 48h. **C** Relative ratio of non-12-OH BAs to 12-OH BAs in ZKY group compared with HFD group at 0 h and 48 h. **D** Relative ratio of unconjugated BAs to corresponding conjugated BAs in ZKY group compared with HFD group at 0 h and 48 h. **E** Design of ex vivo co-culture experiment 2. **F** Relative expression of fatty acid degradation-related genes (*n* = 3). All data are shown as the mean ± SEM (*n* = 3); difference between groups are measured by one-way ANOVA followed by Tukey’s multiple comparison. ^#^*p* < 0.05 (PA/OA Model *vs.* Con); *p < 0.05 (ZKYB_BA *vs.* PA/OA Model)

To determine whether changes in bile acids, especially the ratio of unconjugated BAs, are mediated by GM, we conducted an ex vivo co-culture experiment (Fig. 7A). The fresh cecal contents of HFD-fed and ZKY-intervened mice were collected and the cecal bacteria was then isolated and cultured in GB medium containing fresh bile juice obtained from 4-week-old C57BL/6 J wild type mice for 48 h. The changes in bile acids were then measured and analyzed. As the PLS-DA plot indicated, after 48 h of culture, the bile acid composition of HFD group and ZKY group showed a significant difference (Fig. 7B). Consistent with previous results, the relative proportion of non-12-OH bile acids in the ZKY group showed an increasing trend after 48 h (Fig. 7C). Similarly, at 48 h, the ratio of unconjugated BAs to conjugated BAs in ZKY group was almost twice that of HFD group, which is also consistent with the previous conclusion (Fig. 7D). What’s more, except for βMCA and TβMCA, the ratio of unconjugated BAs to their corresponding conjugated BAs all showed an upward trend at 48 h, which means that the GM intervened by ZKY promoted the deconjugation of conjugated BAs, and then turned into the corresponding unconjugated BAs.

We next studied whether the BAs metabolized by ZKY-altered bacteria contribute to the improvement of metabolism (Fig. 7E). Our results showed that compared with the BAs metabolized by control bacteria, BAs metabolized by ZKY-altered bacteria increased the gene expression of *Ehhadh* and *Hadha* in AML-12 cell, which is consistent with the results of hepatic transcriptome (Fig. 7F). The expression of *Pparα* also showed an increased trend in BAs metabolized by ZKY-altered bacteria than by control bacteria. This finding suggests that ZKY intervention might regulate liver fatty acid metabolism through modulating gut microbiota composition and BAs profile.

## Discussion

**Fig. 8:**
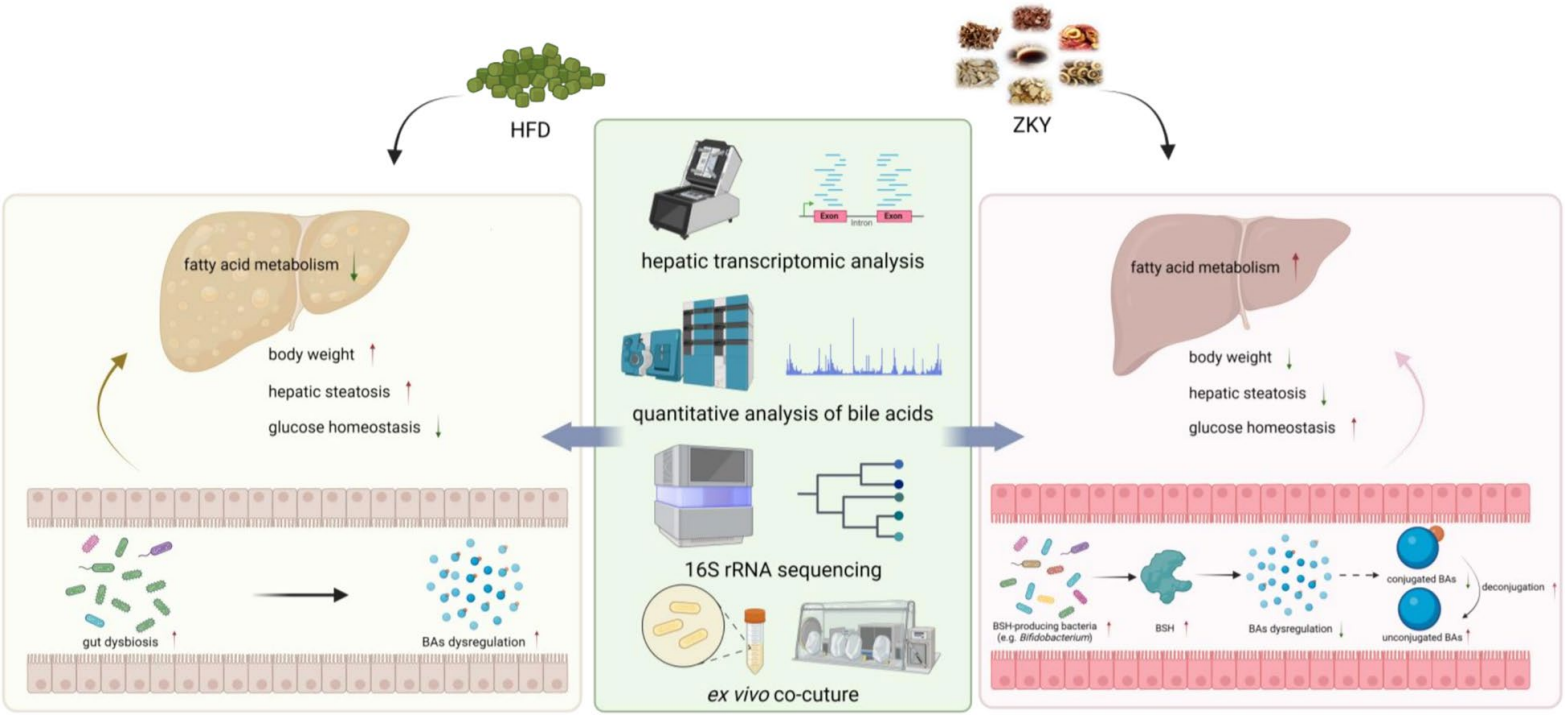
ZKY alleviates high-fat diet-induced metabolic disorders through modulating gut microbiota-bile acids axis

In recent years, although many underlying mechanisms need to be further elucidated, the crosstalk between GM and BAs is still regarded as an important target for improving metabolism and has received increasing attention from researchers. In our present study, we demonstrated that ZKY could reduce weight gain, alleviate hepatic steatosis and improve blood glucose homeostasis in HFD-fed mice. This effect of ZKY was associated with the elevating ratio of unconjugated to conjugated BAs caused by GM-derived increase of BSH expression, which promoting fatty acid degradation (Fig. 8). This finding provides an evidence for the effect of regulation of gut microbiota-bile acid balance on metabolic homeostasis.

The change of GM is an important factor affecting host metabolism. In our study, *f_Lachnospiraceae* is most notable for its high abundance and significant differences between all three groups*.* Members of *Lachnospiraceae* are reported as producers of short-chain fatty acids (SCFAs), while SCFA-mediated activation of GPR43 could suppress insulin signalling in adipocytes, which inhibits fat accumulation and promotes the metabolism of lipid and glucose [32, 33]. Therefore, members of *Lachnospiraceae* are often regarded as beneficial to metabolism. For instance, some studies noted that *Blautia* from *Lachnospiraceae* family is inversely associated with obesity and T2DM [34, 35]. However, it has also been reported that the abundance of *Lachnospiraceae* is positively correlated with metabolic disorders [36], which is consistent with our result that most members of *Lachnospiraceae* showed a decreasing trend after ZKY intervention. Another family that had a significant difference between the ZKY group and the HFD group was *f_Erysipelotrichaceae.* The metabolic functions of this family remain to be controversial among different studies [37–40]. In our study, the intervention of ZKY led to a dramatic increasing of *f_Erysipelotrichaceae* and some genera like *g_Dubosiella* and *g_Ileibacterium* in this family, which provides an evidence for their metabolic benefits.

BAs have been recognized as important signaling molecules that regulate body metabolism and the synthesis, transformation and metabolism of BAs have been extensively studied [41]. Primary BAs are synthesized in the liver by cholesterol in two different routes: classical pathway and alternative pathway [11]. Among them, the classical pathway mainly produces 12-OH primary BAs, while the alternative pathway mainly produces non-12-OH primary BAs [42].The change in the ratio of 12-OH BAs to non-12-OH BAs is closely related to metabolic homeostasis [43]. There have been reported that the activation of the alternative pathway had beneficial effects on glucose and lipid metabolism [44, 45]. Similarly, studies have found that the ratio of 12-OH/non- 12-OH BAs is positively associated with hepatic steatosis in obese mice and rats [46, 47]. Meanwhile, the elevated ratio have also been observed in clinical patients with obesity and T2DM [48]. It has been reported that reducing the ratio of 12-OH/non-12-OH BAs by knockout of CYP8B1 could lead to the decrease in weight gain and improvement of glucose tolerance and hepatic steatosis in western diet-fed mice [49]. Consistent with these studies, in our result, the ratio of 12-OH/non-12-OH BAs showed an increasing trend in HFD group and significantly decreased in ZKY group in serum. The same conclusion could also be obtained from ex vivo co-culture. These results suggest that the increasing proportion of non-12-OH BAs might be one of the reasons causing therapeutic effect of ZKY.

In our study, the increase of unconjugated BAs, especially in serum, was the most significant change found in the BA profile after ZKY intervention. Unconjugated BAs are produced in response to BSH in the intestine, and BSH-related genera mainly include *Bacteroides*, *Clostridiuum*, *Lactobacillus*, *Bifidobacterium*, *Listeria*, *Enterococcus*, *Blautia*, *Roseburia*, etc. [11, 31]. Among these genera, *Bifidobacterium* was the most notable because it showed a significant positive correlation with almost all unconjugated BAs in correlation analysis, and it was also the only genus which demonstrated an increased relative abundance in the ZKY group compared with HFD group in 16S rRNA sequencing. *Bifidobacteria* are widely recognized as probiotics that are beneficial to the metabolic health of the host [50]. Many studies have shown that *Bifidobacterium* intervention can improve HFD-induced obesity, as well as the inflammation and insulin resistance [51, 52]. What’s more, our study proved that ZKY can directly promote the growth of *Bifidobacterium*, but not *Lactobacillus*, another probiotic and BSH-producing genus. These findings further indicates that *Bifidobacterium* may be the critical genus for ZKY to increase the expression of BSH and to improve metabolism. However, except for *Bifidobacterium*, we noted that the abundance of other BSH-producing genera did not increase in the ZKY group, and some related genera were not found in our 16S rRNA sequencing results. This may be due to these BSH-related genera were detected as unclassified or unknown. The uncertainty of sequencing results prompted us to perform qPCR to directly quantify fecal BSH. The results showed that ZKY intervention did increase the copy number of of BSH.

It is worth noting that whether increasing the ratio of unconjugated BAs to conjugated BAs is beneficial to metabolism is inconclusive. In some studies, the decreasing ratio of unconjugated BAs to conjugated BAs has been thought to reduce cholesterol levels and lipid accumulation of the host [44, 53–55]. These findings all mentioned a common mechanism that the decreasing of BSH was shown to increase the proportion of conjugated BAs in intestine, thereby inhibiting the intestinal FXR signaling pathway, thus increasing the level of BA synthesis in the liver, and decreasing liver cholesterol levels [56]. However, opposite results have been observed in other studies that the improvement of lipid metabolism is accompanied by an increase in BSH-related bacteria as well as the proportion of unconjugated BAs [57, 58]. One possible hypothesis is that because unconjugated BAs are more hydrophobic than conjugated BAs, and increase in the proportion of the former in the intestine reduces the intestinal cholesterol solubility and absorption, thereby reducing serum cholesterol level [59]. What’s more, some unconjugated BAs have been separately reported to have metabolic benefits. In addition to HDCA, which has been widely reported to be beneficial to lipid metabolism [12, 13], HCA has also been found to upregulate GLP-1 secretion and production in enteroendocrine cells through simultaneously activating TGR5 and inhibiting intestinal FXR, and thereby improve glucose homeostasis [60]. Besides, UDCA has been reported to activate the SIRT1-PGC1α signaling pathway and reduced sterol regulatory element-binding protein-1 (SREBP-1), thus improve lipid metabolism and reduce body weight of obesity mice [61]. Consistently, in our studies, these mentioned bile acids all showed significantly upward trends in ZKY group compared with HFD group. Therefore, despite the functional controversies, the increase in the proportion of unconjugated BAs is still considered to be an important factor in the efficacy of ZKY.

After confirming the regulation of the GM-BA axis by ZKY, how does the increase of unconjugated BAs lead to the improvement of phenotype is also a very important question. Our ex vivo co-culture experiments indicated that compared with BAs that were not regulated by ZKY, the increase of unconjugated BAs could upregulate the expression of fatty acid oxidation-related genes such as *Ehhadh*, *Hadha*, and *Ppara*, which was also consistent with the transcriptome data that the fatty acid degradation and PPAR signaling pathway were upregulated in ZKY group. Our and other previous studies on HDCA [9, 10] showed that HDCA could improve lipid metabolism by directly regulating this pathway, indicating that fatty acid oxidation may be one of the key pathways for BAs to improve metabolism.

In this study, the effects of ZKY on attenuating HFD-induced metabolic disorders were not dose-dependent. This might be due to TCM formulas have the characteristics of multi-component, multi-target, and multi-pathway, which lead to the complexity of their therapeutic mechanism. Additionally, in this study we didn’t explore the effect of specific chemical components in ZKY. For example, we speculate that the growth of *Bifidobacterium* could be promoted by various components in ZKY. For instance, ZKY contains a variety of terpenoids and flavone, which have been reported to improve lipid metabolism by regulating gut microbiota [62, 63]. Therefore, further work will focus on the investigation of the specific mechanisms of action of various herbal components in ZKY and their interactions.

## Conclusion

Our study reveals that ZKY could attenuate metabolic disorder in HFD-fed mice. The results of 16S rRNA sequencing, hepatic transcriptome, BA profile detection and ex vivo co-culture suggest that the therapeutic efficacy of ZKY relies on changing the composition of GM, resulting in the upregulation of BSH expression, which promotes the deconjugation of bile acids, increases the proportion of unconjugated BAs, upregulates fatty acid degradation pathway and thus improves the glucose and lipid metabolism of the host. These findings provide an evidence to explain the metabolic beneficial effects of ZKY and further studies are needed to elucidate the key herbs and the specific compounds through which ZKY exerts its effects.

## Supplementary Information

Additional file 1

## Data Availability

The data associated with this study can be obtained from the corresponding author upon reasonable request.

## Abbreviations

ALT: Alanine aminotransferase
AST: Aspartate aminotransferase
AUC: Area under glycemic curve
BA: Bile acid
BSH: Bile salt hydrolase
FXR: Farnesoid X receptor
GM: Gut microbiota
HDL-c: High density lipoprotein-cholesterol
HFD: High-fat diet
H&E: Hematoxylin and eosin
IPGTT: Intraperitoneal glucose tolerance test
LDL-c: Low density lipoprotein-cholesterol
MAFLD: Metabolic dysfunction -associated fatty liver disease
OTU: Operational taxonomic unit
PCoA: Principal coordinate analysis
PICRUSt2: Phylogenetic investigation of communities by reconstruction of unobserved states
PLS-DA: Partial least squares-discriminant analysis
TC: Total cholesterol
TCM: Traditional Chinese Medicine
TG: Triglyceride
T2DM: Type 2 diabetes mellitus
ZKY: Zhi-Kang-Yin
