# Supplementary material for: Zhi-Kang-Yin formula attenuates high-fat diet-induced metabolic disorders through modulating gut microbiota-bile acids axis in mice
Source: Chin Med. 2024 Oct 18;19:145. doi: 10.1186/s13020-024-01021-w (PMC11490013; doi:10.1186/s13020-024-01021-w)
Supplement: Supplementary file 1 — Additional file 1 [file 13020_2024_1021_MOESM1_ESM.doc]

Supplementary material

Preparation of ZKY decoction and chemical components Identification

Quantified prescription granules of each herb in ZKY were provided by Hunan University of Traditional Chinese Medicine. Granules of each herb were mixed with one portion each to prepare ZKY decoction which concentration was 1.5 g/ml. Then part of the solution was diluted to 0.75g/ml for different groups of animal experiment

The main chemical components in ZKY decoction were detected by UPLC-MS. The chemical components of ZKY were analyzed by using ultra high performance liquid chromatography-Q exactive hybrid quadrupole orbitrap high-resolution accurate mass spectrometer (UHPLC-Q-Orbitrap HRMS, Thermo Fisher Scientific Inc., Grand Island, NY, USA). A Waters ACQUITY UPLC BEH C_18_ column (2.1*100 mm, 1.7 μm) was employed. The mobile phases consisted of phase A (methanol) and phase B (0.1% formic acid) at a flow rate of 0.3 ml*min^-1^. The cooling autosampler was set at 10℃ and protected from light, while the column heater was set at 40℃. The following protocol of gradient elution was used: 0~4.0 min (4% A), 4.0~10.0 min (4%~12% A), 10.0~30.0 min by (12%~70% A), 30.0~35.0 min by (70% A), 35.0~38.0 min by (70%~95% A), 38.0~42.5 min by (95% methanol), and 42.5~45.0 min by (4% A). Subsequently, the extract of ZKY solution were injected into the UHPLC-Q-Orbitrap HRMS system equipped with an electrospray ionization source, while Xcalibur 4.1 software was used for data recording and analysis. The electrospray ionization source was operated and optimized in positive and negative ionization mode. The optimized parameters of mass spectrometry included capillary temperature at 320℃, sheath gas (N_2_) at a flow rate of 35 arbitrary units, auxiliary gas (N_2_) at a flow rate of 10 arbitrary units, sweep gas at a flow rate of 0 arbitrary units, spray voltage at 3.5 kV (positive mode) and 3.0 kV (negative mode), S-lens RF level at 50 V, auxiliary gas heater temperature at 300℃, scan mode as Full MS/dd-MS, and including two events: a first level full scan (resolution of 70 000 FWHM) and a data dependent second level scan (resolution of 17 500 FWHM). The scanning range of positive and negative ions both m/z 100~1500 while the collision energy gradients are 10 eV, 20 eV and 40 eV. The components of the ZKY extract were identified by comparing the retention time and molecular ions of acquired peaks with self-built MS spectral databases, commercially available reference standards, reference literature, and ChemicalBook. The results were shown in Fig. S1 and Table S1.

Figure S1


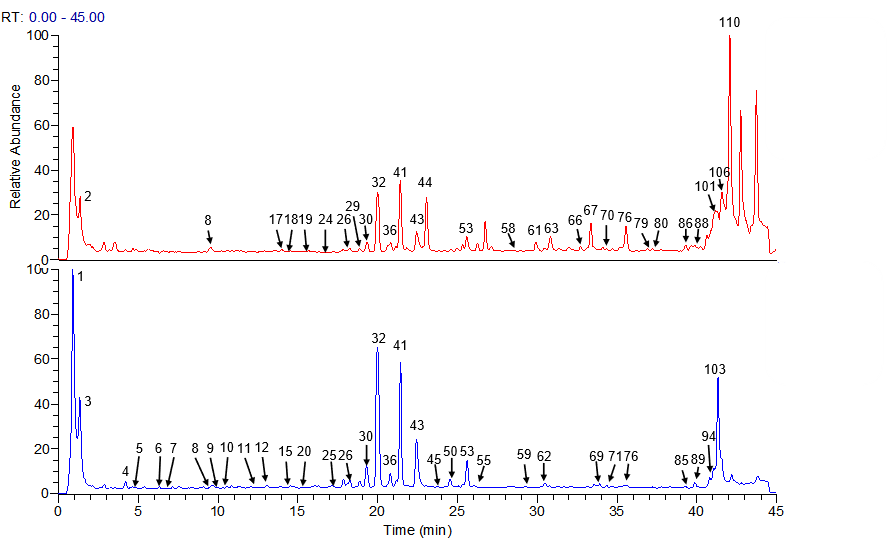


ESI-Negative

ESI-Positive

Figure S1 The UPLC-Q-TOF/MS chromatogram of ZKY decoction.

Figure S2


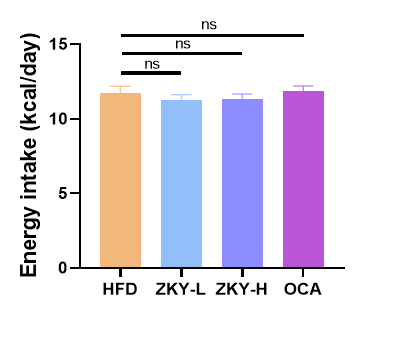


Figure S2. The energy intake of high-fat diet fed mice

Table S1 The identification results of main components of ZKY decoction

| NO. | RT（min） | Adductions | Measured m/z | Expected m/z | Mass error（ppm） | Formula | Identification |
| --- | --- | --- | --- | --- | --- | --- | --- |
| 1 | 0.92 | [M-H]^-^ | 191.0555 | 191.0550 | 2.541 | C_7_H_12_O_6_ | Quinic acid |
| 2 | 1.24 | [M+H]^+^ | 124.0396 | 124.0393 | 2.701 | C_6_H_5_NO_2_ | Nicotinic acid |
| 3 | 1.67 | [M-H]^-^ | 117.0181 | 117.0179 | -1.326 | C_4_H_6_O_4_ | Succinic acid |
| 4 | 4.11 | [M-H]^-^ | 153.0183 | 153.0182 | 0.489 | C_7_H_6_O_4_ | [Protocatechuic acid](https://www.chemsrc.com/en/cas/99-50-3_1188518.html) |
| 5 | 4.22 | [M-H]^-^ | 197.0450 | 197.0444 | 2.640 | C_9_H_10_O_5_ | [Syringic acid](https://www.chemsrc.com/en/cas/530-57-4_403795.html) |
| 6 | 6.34 | [M-H]^-^ | 137.0233 | 137.0233 | -0.515 | C_7_H_6_O_3_ | [3,4-Dihydroxybenzaldehyde](https://www.chemsrc.com/en/cas/139-85-5_1188057.html) |
| 7 | 6.47 | [M-H]^-^ | 353.0885 | 353.0882 | 5.159 | C_16_H_18_O_9_ | [Neochlorogenic acid](https://www.chemsrc.com/en/cas/906-33-2_843309.html) |
| 8 | 9.42 | [M-H]^-^ | 353.0886 | 354.0882 | 5.244 | C_16_H_18_O_9_ | Chlorogenic acid |
| 9 | 9.57 | [M-H]^-^ | 179.0342 | 179.0339 | 1.982 | C_9_H_8_O_4_ | Caffeic acid |
| 10 | 10.54 | [M-H]^-^ | 353.0884 | 355.0882 | 4.904 | C_16_H_18_O_9_ | Cryptochlorogenic acid |
| 11 | 12.61 | [M-H]^-^ | 401.1463 | 401.1442 | 5.102 | C_18_H_26_O_10_ | Icariside F2 |
| 12 | 12.65 | [M-H]^-^ | 335.0783 | 335.0774 | 6.375 | C_16_H_16_O_8_ | 5-O-Caffeoylshikimic acid |
| 13 | 13.13 | [M-H]^-^ | 207.0297 | 207.0294 | 4.541 | C_10_H_8_O_5_ | Fraxetin |
| 14 | 13.41 | [M-H]^-^ | 335.0781 | 335.0774 | 5.808 | C_16_H_16_O_8_ | 6-O-Caffeoylshikimic acid |
| 15 | 14.06 | [M-H]^-^ | 367.1040 | 367.1024 | 4.471 | C_17_H_20_O_9_ | 3-O-Feruloylquinic acid |
| 16 | 14.30 | [M-H]^-^ | 335.0782 | 335.0774 | 6.106 | C_16_H_16_O_8_ | 7-O-Caffeoylshikimic acid |
| 17 | 14.56 | [M+H]^+^ | 595.1664 | 595.1665 | 1.064 | C_27_H_30_O_15_ | Luteolin 7-rutinoside |
| 18 | 14.78 | [M+H]^+^ | 195.0654 | 195.0652 | 1.101 | C_10_H_10_O_4_ | Ferulic acid |
| 19 | 15.45 | [M+H]^+^ | 625.1768 | 625.1763 | 0.814 | C_28_H_32_O_16_ | 4'-O-Methyllucenin II |
| 20 | 15.47 | [M-H]^-^ | 623.1633 | 623.1612 | 4.283 | C_28_H_32_O_16_ | Narcissoside |
| 21 | 15.85 | [M+FA-H]^-^ | 561.1260 | 561.1244 | 3.846 | C_25_H_24_O_12_ | Isochlorogenic acid B |
| 22 | 15.92 | [M-H]^-^ | 741.2268 | 741.2237 | 4.256 | C_33_H_42_O_19_ | Naringenin-7-O-triglycoside |
| 23 | 15.95 | [M-H]^-^ | 623.1631 | 623.1623 | -1.735 | C_28_H_32_O_16_ | Isorhamnetin-3-O-robinobioside |
| 24 | 16.11 | [M+H]^+^ | 471.2019 | 471.1988 | 1.116 | C_26_H_30_O_8_ | Limonin |
| 25 | 17.12 | [M-H]^-^ | 595.1681 | 595.1657 | 3.937 | C_27_H_32_O_15_ | Neoeriocitrin |
| 26 | 18.04 | [M-H]^-^ | 463.1256 | 463.1235 | 4.582 | C_22_H_24_O_11_ | Hesperetin 7-O-glucoside |
| 27 | 18.11 | [M-H]^-^ | 537.1049 | 537.1028 | 4.036 | C_27_H_22_O_12_ | Salvianolic acid I |
| 28 | 18.93 | [M-H]^-^ | 609.1477 | 609.1450 | 4.431 | C_27_H_30_O_16_ | Rutin |
| 29 | 18.95 | [M+H]^+^ | 611.1611 | 611.1605 | 0.669 | C_27_H_30_O_16_ | Quercetin 3-O-neohesperidoside |
| 30 | 19.31 | [M+H]^+^ | 419.1339 | 419.1335 | 0.671 | C_21_H_22_O_9_ | Natsudaidain |
| 31 | 19.77 | [M+H]^+^ | 463.1238 | 463.1233 | 0.760 | C_22_H_22_O_11_ | Diosmetin-7-O-beta-D-glucopyranoside |
| 32 | 19.99 | [M-H]^-^ | 579.1730 | 579.1708 | 3.795 | C_27_H_32_O_14_ | Naringin |
| 33 | 20.28 | [M-H]^-^ | 417.0838 | 417.0816 | 5.219 | C_20_H_18_O_10_ | Salvianolic acid D |
| 34 | 20.63 | [M-H]^-^ | 463.1257 | 463.1235 | 4.712 | C_22_H_24_O_11_ | hesperetin 5-O-glucoside |
| 35 | 20.63 | [M-H]^-^ | 537.1049 | 537.1028 | 3.924 | C_27_H_22_O_12_ | Salvianolic acid H |
| 36 | 20.77 | [M-H]^-^ | 301.0709 | 301.0707 | 0.848 | C_16_H_14_O_6_ | Hesperetin |
| 37 | 21.16 | [M+H]^+^ | 195.0654 | 195.0652 | 1.254 | C_10_H_10_O_4_ | Isoferulic acid |
| 38 | 21.17 | [M-H]^-^ | 359.0781 | 359.0761 | 5.531 | C_18_H_16_O_8_ | Rosmarinic acid |
| 39 | 21.25 | [M-H]^-^ | 187.0970 | 187.0965 | 2.643 | C_9_H_16_O_4_ | Azelaic acid |
| 40 | 21.35 | [M-H]^-^ | 537.1052 | 537.1028 | 4.594 | C_27_H_22_O_12_ | Lithospermic acid |
| 41 | 21.42 | [M-H]^-^ | 609.1836 | 609.1814 | 3.601 | C_28_H_34_O_15_ | Hesperidin |
| 42 | 22.46 | [M-H]^-^ | 537.1041 | 537.1028 | 2.434 | C_27_H_22_O_12_ | Salvianolic acid U |
| 43 | 22.46 | [M-H]^-^ | 717.1476 | 717.1450 | 3.666 | C_36_H_30_O_16_ | Salvianolic acid B |
| 44 | 23.00 | [M+Na]^+^ | 441.1522 | 441.1517 | 0.524 | C_22_H_26_O_8_ | DL-Syringaresinol |
| 45 | 23.02 | [M-H]^-^ | 651.1586 | 651.1556 | 4.691 | C_29_H_32_O_17_ | 3',3,5-Hydroxy-4',5'-methoxyflavone-O-glucoside-orhamnoside |
| 46 | 23.72 | [M-H]^-^ | 537.1054 | 537.1028 | 4.948 | C_27_H_22_O_12_ | Salvianolic acid T |
| 47 | 23.92 | [M-H]^-^ | 301.0360 | 301.0343 | 5.750 | C_15_H_10_O_7_ | Quercetin |
| 48 | 24.20 | [M-H]^-^ | 373.0938 | 373.0918 | 5.243 | C_19_H_18_O_8_ | 5,7-Hydroxy-3',4',5',6-tetramethoxyflavone |
| 49 | 24.50 | [M-H]^-^ | 493.1154 | 493.1129 | 5.002 | C_26_H_22_O_10_ | Salvianolic acid C |
| 50 | 24.54 | [M-H]^-^ | 271.0617 | 271.0601 | 5.719 | C_15_H_12_O_5_ | Naringenin |
| 51 | 24.86 | [M-H]^-^ | 711.2891 | 711.2884 | -8.611 | C_34_H_48_O_16_ | Nomilinic acid glucoside |
| 52 | 25.07 | [M-H]^-^ | 285.0411 | 285.0394 | 5.913 | C_15_H_10_O_6_ | Aureusidin |
| 53 | 25.53 | [M-H]^-^ | 493.1154 | 493.1129 | 5.002 | C_26_H_22_O_10_ | Salvianolic acid A |
| 54 | 25.60 | [M-H]^-^ | 593.1884 | 593.1882 | 3.267 | C_28_H_34_O_14_ | Didymin |
| 55 | 26.02 | [M-H]^-^ | 301.0724 | 301.0707 | 5.631 | C_16_H_14_O_6_ | Salvianolic acid F |
| 56 | 27.55 | [M-H]^-^ | 269.0461 | 269.0444 | 6.282 | C_15_H_10_O_5_ | Apigenin |
| 57 | 28.22 | [M+Na]^+^ | 323.1257 | 323.1254 | 1.082 | C_18_H_20_O_4_ | Salvianonol |
| 58 | 28.53 | [M+H]^+^ | 728.3984 | 728.3975 | 0.944 | C_36_H_53_N_7_O_9_ | Citrusin III |
| 59 | 29.23 | [M-H]^-^ | 329.1402 | 329.1384 | 5.711 | C_19_H_22_O_5_ | 13R-14R-Hydroxy-anhydride of 16R cryptotanshinone |
| 60 | 29.55 | [M+FA-H]^-^ | 843.4771 | 843.4742 | 4.087 | C_42_H_70_O_14_ | HydroxyNepasaikosaponin A isomer |
| 61 | 29.91 | [M+H]^+^ | 373.1284 | 373.1278 | 0.564 | C_20_H_20_O_7_ | Isosinensetin |
| 62 | 30.20 | [M+FA-H]^-^ | 843.4772 | 843.4742 | 4.230 | C_42_H_70_O_14_ | HydroxyNepasaikosaponin A |
| 63 | 30.83 | [M+H]^+^ | 373.1284 | 373.1278 | 0.564 | C_20_H_20_O_7_ | Sinensetin |
| 64 | 31.24 | [M+H]^+^ | 183.0807 | 183.0804 | 1.521 | C_13_H_10_O | 7-Phenyl-2-heptene-4,6-diyn-1-ol |
| 65 | 32.60 | [M+Na]^+^ | 335.0896 | 335.0890 | 1.896 | C_18_H_16_O_5_ | Tanshindiol C |
| 66 | 32.66 | [M+H]^+^ | 545.3456 | 545.3473 | -3.008 | C_32_H_48_O_7_ | Alisol M 23-acetate |
| 67 | 33.35 | [M+H]^+^ | 403.1391 | 403.1387 | 0.808 | C_21_H_22_O_8_ | Nobiletin |
| 68 | 33.45 | [M+H]^+^ | 341.1384 | 341.1384 | 0.234 | C_20_H_20_O_5_ | Dihydromethyl Tanshinonate |
| 69 | 33.90 | [M-H]^-^ | 329.2341 | 329.2323 | 5.617 | C_18_H_34_O_5_ | Pinellic acid isomer |
| 70 | 34.12 | [M+H]^+^ | 433.1498 | 433.1493 | 1.019 | C_22_H_24_O_9_ | 3,5,6,7,8,3’,4’-Heptamethoxyflavone |
| 71 | 34.31 | [M-H]^-^ | 329.2341 | 329.2323 | 5.526 | C_18_H_34_O_5_ | Pinellic acid |
| 72 | 34.31 | [M+FA-H]^-^ | 971.5247 | 971.5216 | 3.761 | C_48_H_78_O_17_ | Saikosaponin C |
| 73 | 34.68 | [M+H]^+^ | 309.1123 | 309.1121 | 0.435 | C_19_H_16_O_4_ | Tanshinone IIB |
| 74 | 35.22 | [M-H]^-^ | 327.1246 | 327.1227 | 5.716 | C_19_H_20_O_5_ | 1S-Hydroxy-anhydride of 16R cryptotanshinone |
| 75 | 35.44 | [M+H]^+^ | 471.3469 | 471.3469 | -0.056 | C_30_H_46_O_4_ | Alisol H |
| 76 | 35.55 | [M+H]^+^ | 373.1284 | 373.1282 | 0.645 | C_20_H_20_O_7_ | Tangeretin |
| 77 | 35.62 | [M+H]^+^ | 337.1411 | 337.1434 | -6.898 | C_21_H_20_O_4_ | Danshenxinkun D |
| 78 | 36.18 | [M+H]^+^ | 279.1018 | 279.1016 | 0.964 | C_18_H_14_O_3_ | Dihydrotanshinone I |
| 79 | 36.89 | [M+H]^+^ | 487.3424 | 487.3418 | 1.250 | C_30_H_46_O_5_ | Alisol C |
| 80 | 37.17 | [M+Na]^+^ | 551.3350 | 551.3343 | 1.287 | C_32_H_48_O_6_ | Alisol C 23-acetate |
| 81 | 37.18 | [M+H]^+^ | 489.3582 | 489.3575 | 1.490 | C_30_H_48_O_5_ | 13β,17β-epoxyalisol B |
| 82 | 37.67 | [M+Na]^+^ | 303.0994 | 303.0992 | 0.773 | C_18_H_16_O_3_ | 1,2,15,16-Tetrahydrotanshinquinone |
| 83 | 38.02 | [M+H]^+^ | 315.1567 | 315.1591 | -7.443 | C_19_H_22_O_4_ | Neocryptotanshinone |
| 84 | 38.32 | [M+H]^+^ | 471.3475 | 471.3469 | 1.302 | C_30_H_46_O_4_ | 16,23-Oxidoalisol B |
| 85 | 39.41 | [M-H]^-^ | 821.4700 | 821.4688 | 2.248 | C_44_H_70_O_14_ | 2''-O-Acetylsaikosaponin A |
| 86 | 39.49 | [M+H]^+^ | 531.3686 | 531.3680 | 1.081 | C_32_H_50_O_6_ | Alisol S 23-acetate |
| 87 | 39.69 | [M+H]^+^ | 471.3476 | 471.3469 | 1.429 | C_30_H_46_O_4_ | Alisol U |
| 88 | 39.87 | [M+H]^+^ | 455.3523 | 455.3520 | 0.787 | C_30_H_46_O_3_ | Alisol I |
| 89 | 39.88 | [M+FA-H]^-^ | 825.4664 | 825.4636 | 4.037 | C_42_H_68_O_13_ | Saikosaponin A |
| 90 | 40.04 | [M+H]^+^ | 297.1488 | 297.1485 | 0.838 | C_19_H_20_O_3_ | Cryptotanshinone |
| 91 | 40.06 | [M-H]^-^ | 821.4691 | 821.4688 | 1.141 | C_44_H_70_O_14_ | 3''-O-Acetylsaikosaponin A |
| 92 | 40.40 | [M-H]^-^ | 821.4721 | 821.4688 | 4.707 | C_44_H_70_O_14_ | 6''-O-Acetylsaikosaponin A |
| 93 | 40.57 | [M+FA-H]^-^ | 663.4135 | 663.4108 | 4.787 | C_36_H_57_O_8_ | Prosaikogenin D |
| 94 | 40.78 | [M+FA-H]^-^ | 825.4666 | 825.4636 | 4.182 | C_42_H_68_O_13_ | Saikosaponin D |
| 95 | 40.81 | [M+FA-H]^-^ | 809.4717 | 809.4687 | 4.394 | C_42_H_68_O_12_ | Saikosaponin M |
| 96 | 40.85 | [M-H]^-^ | 821.4692 | 821.4688 | 1.287 | C_44_H_70_O_14_ | 2''-O-Acetylsaikosaponin D |
| 97 | 40.88 | [M+FA-H]^-^ | 663.4134 | 663.4108 | 4.969 | C_36_H_57_O_8_ | Prosaikogenin G |
| 98 | 40.99 | [M-H]^-^ | 821.4697 | 821.4688 | 1.883 | C_44_H_70_O_14_ | 3''-O-Acetylsaikosaponin D |
| 99 | 41.06 | [M+FA-H]^-^ | 825.4664 | 825.4636 | 3.964 | C_42_H_68_O_13_ | Saikosaponin b2 |
| 100 | 41.13 | [M+FA-H]^-^ | 663.4128 | 663.4108 | 3.867 | C_36_H_57_O_8_ | Prosaikogenin F |
| 101 | 41.15 | [M+Na]^+^ | 513.3557 | 513.3550 | 1.294 | C_30_H_50_O_5_ | Alisol A |
| 102 | 41.15 | [M+H]^+^ | 473.3631 | 473.3625 | 1.190 | C_30_H_48_O_4_ | Alisol B |
| 103 | 41.20 | [M-H]^-^ | 505.3546 | 505.3524 | 4.441 | C_30_H_50_O_6_ | 13β,17β-epoxyalisol A |
| 104 | 41.25 | [M+H]^+^ | 515.3738 | 515.3731 | 1.317 | C_32_H_50_O_5_ | Alisol B 23-acetate |
| 105 | 41.46 | [M+H]^+^ | 515.3738 | 515.3731 | 1.317 | C_32_H_50_O_5_ | 11-Deoxy-13β,17β-epoxy-23-acetylalisol B |
| 106 | 41.49 | [M+H]^+^ | 503.3557 | 503.3520 | 7.486 | C_34_H_46_O_3_ | Alismanin A |
| 107 | 41.54 | [M+FA-H]^-^ | 825.4705 | 825.4636 | 8.992 | C_42_H_68_O_13_ | Saikosaponin b1 |
| 108 | 41.60 | [M+H]^+^ | 503.3692 | 503.3731 | -7.690 | C_31_H_50_O_5_ | Alisol T |
| 109 | 41.68 | [M-H]^-^ | 443.3500 | 443.3520 | -4.357 | C_29_H_48_O_3_ | Alismanin B |
| 110 | 42.05 | [M+H]^+^ | 499.3780 | 499.3782 | -0.454 | C_32_H_50_O_4_ | 11-Deoxy-23-acetylalisol B |

Table S2 Formulation of HFD

| Ingredients | Grams |
| --- | --- |
| Casein, Lactic, 30 Mesh | 200.00 g |
| Cystine, L | 3.00 g |
| Lodex 10 | 125.00 g |
| Sucrose, Fine Granulated | 72.80 g |
| Solka Floc, FCC200 | 50.00 g |
| Lard | 245.00 g |
| Soybean Oil, USP | 25.00 g |
| Mixed mineral (S10026B) | 50.00 g |
| Choline Bitartrate | 2.00 g |
| Mixed vitamin (V10001C) | 1.00 g |
| Dye, Blue FD&C #1, Alum. Lake 35-42% | 0.05 g |
| Total | 773.85 |

Table S3 The genes and primer sequences

| Gene | Primer sequence (5' to 3') |
| --- | --- |
| *18s-F* | CCATCCAATCGGTAGTAGCG |
| *18s-R* | GTAACCCGTTGAACCCCATT |
| *Hadha-F* | TGCATTTGCCGCAGCTTTAC |
| *Hadha-R* | GTTGGCCCAGATTTCGTTCA |
| *Ppara-F* | CTATAATTTGCTGTGGAGATCGGC |
| *Ppara-R* | GGATGGTTGCTCTGCAGGT |
| *Ehhadh-F* | TGGCTCTAACCGTATGGTCC |
| *Ehhadh-R* | CTATGATCCGCCTCTGCAA |
